# A Sedentary Lifestyle Changes the Composition and Predicted Functions of the Gut Bacterial and Fungal Microbiota of Subjects from the Same Company

**DOI:** 10.1007/s00284-023-03480-0

**Published:** 2023-10-13

**Authors:** Longwei Xu, Wenkun Li, Lu Ling, Ziran Zhang, Zilu Cui, Jiang Ge, Yun Wang, Qianlong Meng, Yadan Wang, Kuiliang Liu, Jun Zhou, Fanxin Zeng, Jing Wang, Jing Wu

**Affiliations:** 1 Department of Gastroenterology, Beijing Shijitan Hospital, Capital Medical University, Beijing, 100038 China; 2 Department of Gastroenterology, National Clinical Research Center for Digestive Diseases, Beijing Friendship Hospital, Capital Medical University, Beijing, 100050 China; 3 Department of Clinical Laboratory, Beijing Chaoyang Hospital, Capital Medical University, Beijing, China; 4 Department of Gastroenterology, School of Clinical Medicine, Peking University Ninth, Beijing, 100038 China; 5 Department of Clinical Research Center, Dazhou Central Hospital, Sichuan, China; 6 No. 95, Yongan Road, Xicheng District, Beijing, 100050 China

## Abstract

A sedentary lifestyle affects the diversity and composition of the gut microbiota, but previous studies have mainly focused on bacteria instead of fungi. Here, we compared both the fecal bacterial and fungal microbiota compositions and functions in sedentary persons and controls. Subjects from the China Railway Corporation, including 99 inspectors and 88 officials, were enrolled in our study. Fecal microbiota communities were analyzed using 16S rRNA gene sequencing for bacteria and ITS sequencing for fungi. We found that the diversity of the gut microbiota of the sedentary group was significantly lower than that of the control group (*P* < 0.05). The sedentary group had a higher abundance of Firmicutes, a lower abundance of Actinobacteria and Proteobacteria and a higher abundance of Ascomycota, and a lower abundance of Basidiomycota. Furthermore, functional prediction analysis of the fungal microbiota revealed more L-tryptophan degradation to 2-amino-3-carboxymuconate semialdehyde, more phospholipid remodeling (phosphatidylethanolamine, yeast), and more L-tyrosine degradation I, as well as less pentose phosphate pathway (non-oxidative branch), less adenosine nucleotide biosynthesis and less L-valine biosynthesis in the sedentary group (*P* < 0.05). Thus, a sedentary lifestyle changes the composition and function of the gut microbiota. It may change the pentose phosphate pathway (non-oxidative branch), nucleic acid and amino acid biosynthesis and phospholipid metabolism in fungi.

**Supplementary Information:**

The online version contains supplementary material available at 10.1007/s00284-023-03480-0.

## Introduction

There are a large number of microorganisms inhabiting in the human gastrointestinal tract, including bacteria, fungi, viruses, parasites, archaea, protist, and worms. These microorganisms maintain a symbiotic relationship with humans by providing energy and nutrition [1], catabolizing compounds [2], regulating immunity [3], preventing pathogen overgrowth [4], regulating intestinal cell endocrine function [5], synthesizing neurotransmitters [6], and so on. Dysbiosis of the microbiota composition can impact host homeostasis in the form of overgrowth, by producing harmful catabolites and inducing inflammation. Multiple diseases such as gastrointestinal diseases [7], neurological diseases [8], lung diseases, metabolic diseases, liver diseases [9], and cardiovascular diseases [10] are associated with the gut microbiota.

Factors including host genetic characteristics, host immune response, diet, infection, exercise, and environment affect the gut microbiota [11]. Previous studies have mainly focused on age, gender, diet, the environment, antibiotics, and probiotics. At present, the human lifestyle has changed considerably, and an increasing number of people have adopted a sedentary lifestyle. Sedentary behavior and a lack of physical exercise can affect the composition, diversity, abundance, and function of the gut microbiota [2, 11, 12], causing many health problems, such as obesity, diabetes, cancer, and heart disease [13]. Previous studies have mainly focused on bacteria and have rarely involved fungi and viruses. However, the balance of the gut fungal microbiota is inextricably linked to physical conditions, which cannot be ignored.

To explore the impact of a sedentary lifestyle on the composition and predicted function of the gut bacterial and fungal microbiota, we conducted the present study. Our research subjects were all from the China Railway Corporation, including 99 inspectors and 88 officials. According to the characteristics of their work types, officials were classified as the sedentary group, while train ticket inspectors were defined as the control group. Differences in the composition and function of both the gut bacterial and fungal microbiota of the two groups were compared.

## Materials and Methods

### Sampling and Information Collection

Subjects including 99 inspectors and 88 officials from the China Railway Corporation were enrolled in this study. The daily sitting time threshold associated with all-cause mortality (ACM) is 8 h per day [14]. Sedentary lifestyle was considered as daily total walking steps less than 5000 in a study [15]. Officials (who sat at work for 8 h and had fewer than 5000 steps) were classified as the sedentary group, while train ticket inspectors (who were physically active at work) were defined as the control group. The exclusion criteria were as follows: pathology, having undergone gastrointestinal surgery, pregnant, lactating, serious chronic illness (e.g., diabetes, heart failure, cancer, or autoimmune diseases), and taking any antibiotics or probiotics within the three months prior to the sampling dates. The fecal sample was obtained in a non-invasive way, with less pain, lower cost, and risk to subjects, which was collected for analysis of microbiota. Each subject donated a fecal sample, which was frozen immediately after sampling and stored at − 80 °C.

Questionnaires including general information on age, gender, body mass index (BMI), exercise information, and dietary habits were completed by all of the subjects through a face-to-face interview. The BMI was calculated as weight in kilograms divided by height in meters squared. Exercise information collected in our study refers to exercise in the subjects’ spare time after work. The frequency of physical exercise per week was classified as ≤ 1 day, 2–3 days, and ≥ 4 days. The frequency of each diet type was classified as never, occasional, frequent, and daily.

### DNA Extraction, 16S rRNA Gene and ITS Amplification and Sequencing

Total fecal DNA was extracted using QIAamp DNA Stool Mini Kit (Qiagen, Hilden, Germany) according to the manufacturer’s instructions. One percent agarose gel electrophoresis was used to assess the purity of DNA. All DNA samples were quality checked and the concentration was quantified by NanoDrop 2000 spectrophotometers (Thermo Fisher Scientific, Wilmington, DE, USA). The universal primers 338F (5'-ACTCCTACGGGAGGCAGCAG-3') and 806R (5'-GGACTACHVGGGTWTCTAAT-3') were used to amplify the bacterial 16S rRNA gene fragments (V3-V4) under the following amplification conditions: 3 min at 95 °C, 30 s at 95 °C, 30 s at 55 °C, and 45 s at 72 °C for 23 cycles; 10 min at 72 °C, 10 °C until halted by user. The primers ITS1F (CTTGGTCATTTAGAGGAAGTAA) and ITS2R (GCTGCGTTCTTCATCGATGC) were used to amplify the fungal internal transcribed spacer regions 1 (ITS1) by the following amplification conditions: 3 min at 95 °C, 30 s at 95 °C, 30 s at 55 °C, and 45 s at 72 °C for 35 cycles; 10 min at 72 °C, 10 °C until halted by user, with the assistance of ABI GeneAmp 9700 PCR system (Applied Biosystems, Foster City, CA, USA). The amplifications were sequenced on the Illumina MiSeq PE300 sequencing platform (Illumina, San Diego, CA, US).

### Amplification Sequence Processing and Analysis

After demultiplexing, the resulting sequences were merged with FLASH (v1.2.11) and quality filtered with fastp (0.19.6). Then the high-quality sequences were de-noised using DADA2 plugin in the Qiime2 (version 2020.2) pipeline with commended parameters, which obtains single nucleotide resolution based on error profiles within samples. DADA2 denoised sequences are usually called amplicon sequence variants (ASVs). To minimize the effects of sequencing depth on alpha and beta diversity measure, the number of sequences from each sample was rarefied to 4000, which still yielded an average Good’s coverage of 97.90%. Taxonomic assignment of ASVs was performed using the Naive Bayes consensus taxonomy classifier implemented in Qiime2 and bacterial (SILVA 16S rRNA database) and fungal libraries (UNITE database). The functional prediction of the community was completed by conducting a phylogenetic survey of the reconstructed unobserved state 2 (PICRUSt2), a mature bioinformatics technology, according to the previous research [16], under the guidelines at https://github.com/picrust/picrust2/wiki.

### Statistical Analysis

SPSS 25.0, GraphPad Prism 8.0, Cytoscape 3.7.2, STAMP and R version 3.5.1 software were used for statistical analysis and plotting. Continuous variables conforming to normal distribution were recorded as the mean ± standard deviation and were analyzed by Student’s *t* test, while un-normally distributed continuous variables were recorded as the median with interquartile range and were analyzed by the Mann–Whitney *U* test. Categorical variables were reported as percentages and analyzed by Pearson’s chi-square test. The Wilcoxon rank-sum test was used to compare the differences in intestinal microbiota between the two groups. Spearman’s rank correlation analysis was used to explore the correlation between clinical features and the gut microbiota. *P* < 0.05 was considered statistically significant.

The power analysis test of independent sample *T* test was conducted by SPSS 27.0 software to determine the smallest sample size. The smallest sample size was calculated by setting a power value as 0.8, a group size ratio as 1, a population mean difference as 1, a pooled population standard deviation as 2, and a significance level as 0.05.

As gut microbiota composition can be influenced by several factors, such as gender, age, lifestyle, environmental factors, and diet, it’s necessary to conduct a linear regression analysis to explore the influence of sedentary on the gut microbiota. Age, and factors that were different between the two groups, including sitting, gender, and going out with the train, were enrolled in the linear regression analysis. The linear regression analysis was conducted by SPSS 27.0 software. The independent variable was sitting and the dependent variable was simpson index, while the control variables were age, gender, and going out with the train.

## Results

### Characteristics of the Subjects

The power analysis test was conducted to determine the smallest sample size and the smallest sample size was calculated as 64 (Table 1). While the sample size of our study was 88 for the sedentary group and 99 for the control group. The power analysis of the actual sample size (n1 = 88, n2 = 99) shown that the power was 0.924 (Table 2). The average ages of the subjects were 48.60 ± 5.31 years for the sedentary group and 49.01 ± 5.597 years for the control group. Since the majority of railway workers were male, the proportion of males in each group was 84.09% and 98%, respectively. There were no significant differences between the two groups in exercise frequency in their spare time after work or in the intake frequency of some types of food. The rest of the information is shown in Table 3.

**Table 1:** The power Analysis used for calculating the smallest sample size

	Power analysis table						
	N1	N2	Actual power^b^	Test assumptions			
				Power	Std. Dev.^c^	Effect size	Sig
Test for mean difference^a^	64	64	.801	.8	2	.500	.05

^a^Two-sided test

^b^Based on noncentral t-distribution

^c^Group variances are assumed to be equal

The power analysis test was conducted to calculate the smallest sample size

**Table 2:** The power Analysis of the actual sample size

	Power analysis table					
	Power^b^	Test assumptions				
		N1	N2	Std. Dev.^c^	Effect size	Sig
Test for mean difference^a^	.924	88	99	2	.500	.05

^a^Two-sided test

^b^Based on noncentral t-distribution

^c^Group variances are assumed to be equal

A power analysis of the actual sample size (n1 = 88, n2 = 99) was conducted to verify the actual power of this study sample size (Table 2)

**Table 3:** Baseline demographics and clinical characteristics of the study cohort

Characteristic	Sedentariness (*n* = 88)	Control (*n* = 99)	*P*
Age	48.60(5.31)^a^	49.01(5.597)	0.498
Male	74(84.09%)^b^	97(98.0%)	< 0.001
Married	85 (96.59%)	96(96.97%)	0.289
BMI(kg/m2)	27.04(4.69)	26.84(5.02)	0.881
Sitting	70(79.55%)	20(20.20%)	< 0.001
Going out with the train	28(31.82%)	17(17.17%)	0.022
Vegetable intake frequency			0.825
Occasionally	9(10.01%)	15(15.15%)	
Often	39(43.8%)	50(50.51%)	
Everyday	40(46.1%)	31(31.31%)	
Fruit intake frequency			0.128
Occasionally	23(26.14%)	39(39.39%)	
Often	39(44.32%)	35(35.35%)	
Everyday	24(27.27%)	21(21.21%)	
Pickled food intake frequency			0.723
Never	3(3.41%)	3(3.03%)	
Occasionally	62(70.45%)	68(68.69%)	
Often	18(20.45%)	22(22.22%)	
Everyday	3(3.41%)	1(1.01%)	
Red meat intake frequency			0.096
Never Occasionally	0 13(14.77%)	1(1.01%) 27(27.27%)	
Often	66(75%)	57(57.58%)	
Everyday	9(10.23%)	10(10.10%)	
White meat intake frequency			0.229
Occasionally	40(45.45%)	49(49.49%)	
Often	44(50%)	38(38.38%)	
Everyday	1(1.14%)	4(4.04%)	
Exercise frequency			0.606
≤ 1 day/week	27(30.68%)	25(25.25%)	
2–3 day/week	31(35.28%)	39(39.39%)	
≥ 4 day/week	20(22.73%)	20(20.20%)	

Baseline demographics and clinical characteristics of the study cohort. ^a^The numbers in the parentheses represent the standard deviation. ^b^The results were shown as number of the subjects and the relative percentage

### Diversity Analysis of the Gut Microbiota Between the Two Groups

**Fig. 1:**
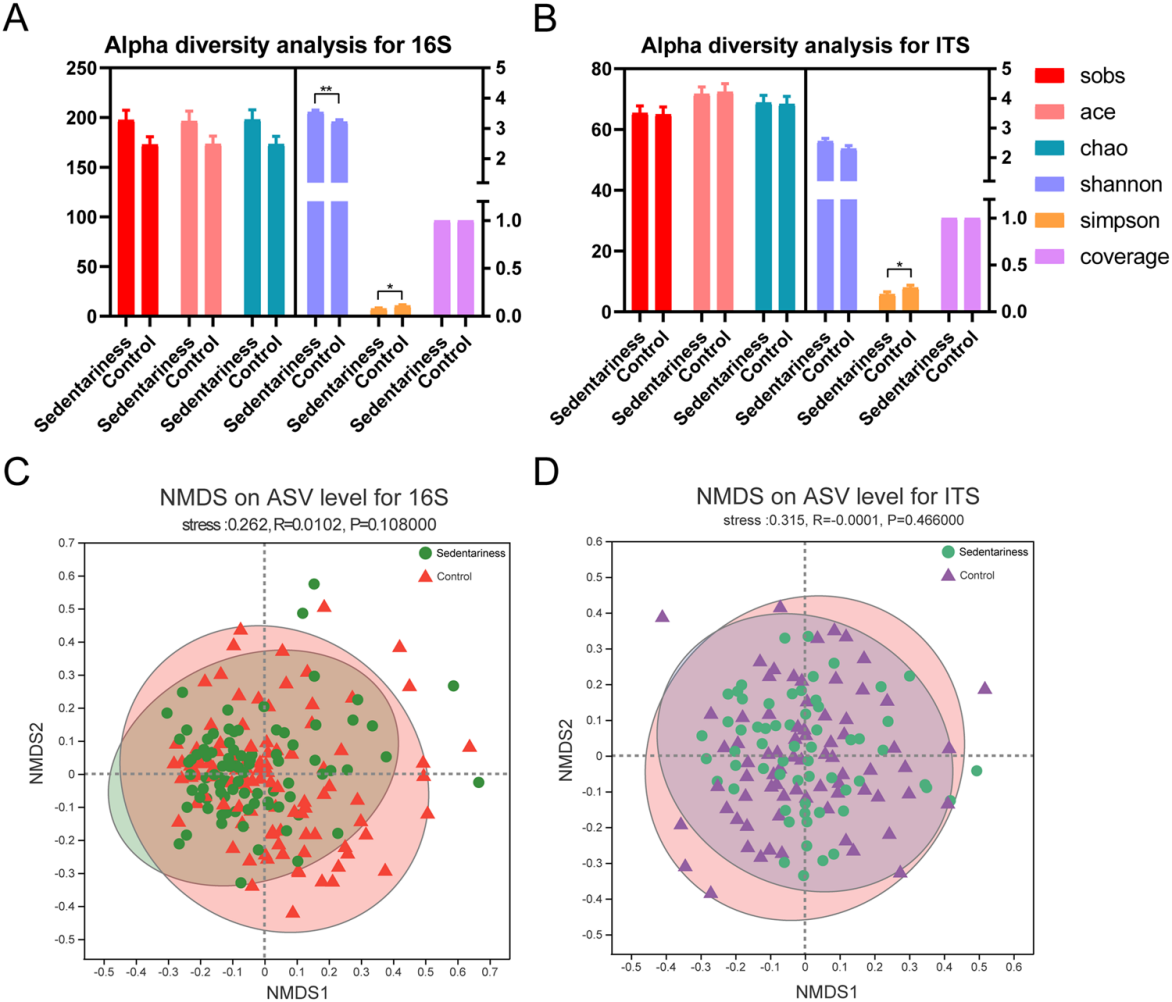
Diversity analysis for gut microbiota. **a** Alpha diversity analysis for the bacterial gut microbiota; **b** Alpha diversity analysis for the fungal gut microbiota; **c** NMDS analysis on ASV level for the bacterial gut microbiota; **d** NMDS analysis on ASV level for the fungal gut microbiota. The higher the similarity between samples, the more concentrated they are in the figure. * *P* < 0.05, ** *P* < 0.01

The rank-abundance curves revealed that species richness and evenness were higher in the bacterial microbiota of the sedentary group, but similar in the fungal microbiota of the two groups (Figure S1A, B). In addition, the numbers of total genera by Pan analysis for 16S and ITS were lower in the sedentary group (Figure S1C, D). There was no difference in the coverage rate between the two groups (Fig. 1a, b), which was approximately 1, indicating that the sequencing depth has covered all the species in the samples. There was no difference in the alpha diversity indices including Ace, Chao, and Sobs, between the two groups. The Shannon index was higher in the sedentary group in 16S rRNA gene sequencing, while there was no difference in ITS sequencing. The Simpson index was significantly lower in the sedentary group both for 16S rRNA gene sequencing and ITS sequencing, indicating that the diversity of the gut microbiota in the sedentary group was lower than that in the control group. Nonmetric multidimensional scaling (NMDS) analysis showed no significant difference in bacterial and fungal community formation between the two groups (Fig. 1c,d).

The microbiota can be influenced by various factors, including age, gender, lifestyle choices, environmental conditions, and dietary habits. As gender and going out with the train were different in the two groups (Table 3), to explore the effects of sitting on the diversity of the gut microbiota, the linear regression analysis including age, gender, and sitting has been conducted. As the result shown (Table 4) that, the *p* value of gender, age, and going out with the train was 0.978, 0.559, and 0.322, respectively, while the *p* value of sitting was 0.008. Which means that the sedentary behavior indeed affects the diversity of gut microbiota, regardless of the factor gender, age, and going out with the train.

**Table 4:** Linear regression analysis of covariate

Coefficient								
Model		Unstandardized coefficient		Standardized coefficient	T value	Significance (P value)	Collinearity Statistics	
		B	Standard error	Beta			Tolerance	VIF
1	Constant	.088	.217		.406	.685		
	Gender	.002	.070	.003	.028	.978	.908	1.102
	Age	− .002	.004	− .055	− .586	.559	.962	1.040
	Setting	.106	.039	.256	2.698	**.008**	.954	1.048
	Going out with the train	.043	.043	.096	.995	.322	.926	1.080

The power analysis test was conducted to calculate the smallest sample size (Table 1)

Linear regression analysis of covariate. The linear regression analysis including sitting, gender, age, and going out with the train has been conducted by SPSS 27.0 software to explore the effects of sitting on the gut microbiota. The independent variable was sitting and the dependent variable was simpson index, while the control variables were age, gender, and going out with the train

### Microbiota Composition of the Two Groups

In 16S rRNA gene sequencing, the Venn plot based on the level of ASV showed that there were 1342 ASVs that were common in the two groups, 5643 special ASVs in the sedentary group and 5609 special ASVs in the control group (Figure S2A), while in ITS sequencing, there were 605 ASVs that were common in the two groups, 1612 special ASVs in the sedentary group and 1943 special ASVs in the control group (Figure S2B).

**Fig. 2:**
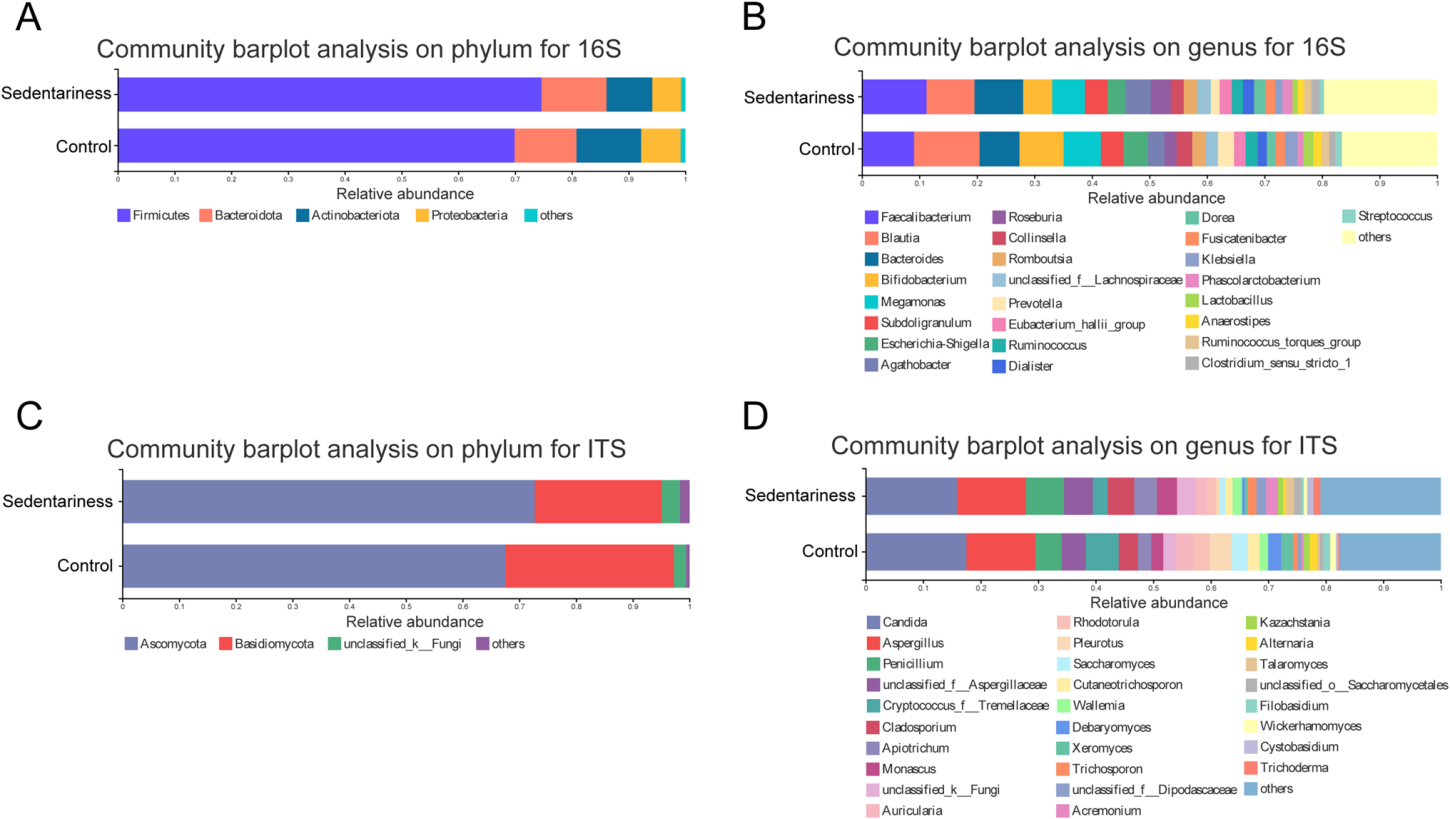
Microbiological composition for the gut microbiota. **a** Community barplot analysis on phylum level for the bacterial gut microbiota; **b** Community barplot analysis on genus level for the bacterial gut microbiota; **c** Community barplot analysis on phylum level the fungal gut microbiota; **d** Community barplot analysis on genus level for the fungal gut microbiota

The fecal bacteria of the sedentary and control groups were both composed of Firmicutes, Bacteroidetes, Actinobacteria, and Proteobacteria (Fig. 2a). The sedentary group had a higher abundance of Firmicutes and a lower abundance of Actinobacteria and Proteobacteria than the control group. At the genus level, the fecal bacteria of the sedentary and control groups were both composed of *Faecalibacterium, Blautia, Bacteroides, Bifidobacterium, Megamonas, Subdoligranulum, Escherichia-Shigella, Agathobacter, Roseburia, Collinsella, Romboutsia,* etc. (Fig. 2b). The fecal fungi of the two groups were both mainly composed of Ascomycota and Basidiomycota at the phylum level (Fig. 2c). The sedentary group had a higher abundance of Ascomycota, and a lower abundance of Basidiomycota. At the genus level, the two groups were both composed of *Candida*, *Aspergillus*, *Penicillium*, *unclassified Aspergillaceae*, *Cryptococcus, Tremellaceae*, *Cladosporium*, *Apiotrichum*, *Monascus*, *Auricularia*, *Rhodotorula*, *Pleurotus*, *Saccharomyces,* etc. (Fig. 2d). The relative abundance of *Candida* in the controls was higher than that in the sedentary group.

### Microbial Difference Analysis Between the Two Groups

**Fig. 3:**
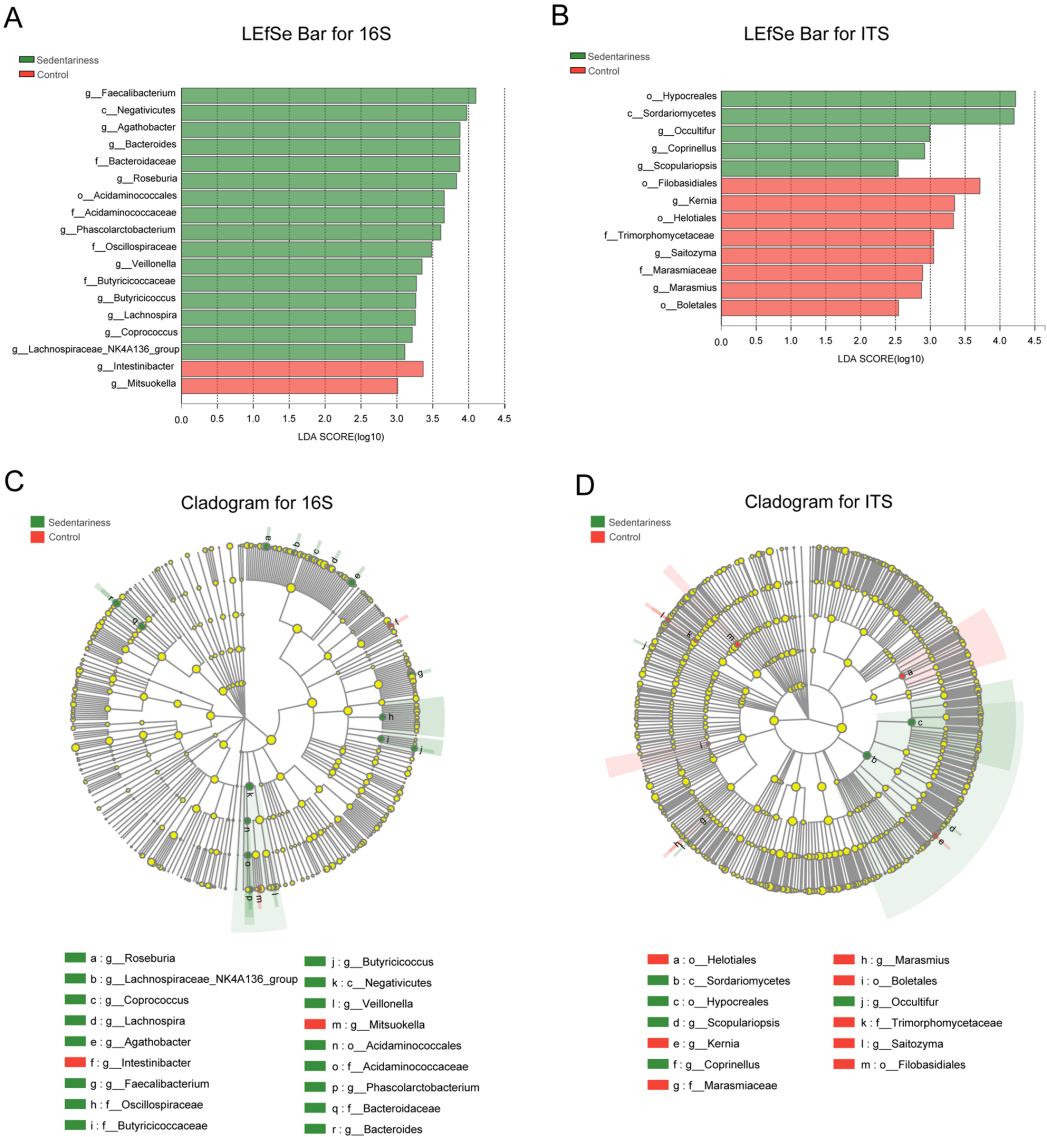
Analysis of Microbiota differences for gut microbiota between the two groups. **a** LDA analysis results for the bacterial gut microbiota; **b** LDA analysis results for the fungal gut microbiota; **c** The cladogram analyzed LEfSe of the bacterial gut microbiota; **d** The cladogram analyzed LEfSe of the fungal gut microbiota

Linear discriminant analysis (LDA) effect size (LEfSe) analysis was used to compare the fecal microbiota between the two groups, with an LDA score cutoff of 3.0 to determine important taxonomic differences between the two groups. The results showed that there were significant differences in the fecal microbiota between the two groups based on LEfSe analysis (Fig. 3a–d). For the bacterial microbiota, the relative abundances of *Faecalibacterium* (*P* = 0.036)*, Negativicutes* (*P* = 0.034)*, Agathobacter* (*P* = 0.009)*, Bacteroidaceae* (*P* = 0.039)*, Bacteroides* (*P* = 0.039)*, Roseburia* (*P* = 0.002), etc., were significantly higher in the sedentary group, while the relative abundances of *Intestinibacter* (*P* = 0.022) and *Mitsuokella* (*P* = 0.002) were significantly higher in the control group (Fig. 3a). For the fungal gut microbiota, the relative abundance of *Hypocreales* (*P* = 0.01), *Sordariomycetes* (*P* = 0.012), *Occultifur* (*P* = 0.033), *Coprinellus* (*P* = 0.03), and *Scopulariopsis* (*P* = 0.025) were significantly higher in the sedentary group. The relative abundances of *Filobasidiales* (*P* = 0.046), *Kernia* (*P* = 0.039), *Helotiales* (*P* = 0.049), *Trimorphomycetaceae* (*P* = 0.023), *Saitozyma* (*P* = 0.023), *Marasmius* (*P* = 0.039), *Marasmiaceae* (*P* = 0.039), etc., were significantly higher in the control group (Fig. 3b). The abundances of certain different species in the LEfSe analysis are shown in Figure S3.

Gender is one of the factors affecting the gut microbiota. The differential bacterial and fungal genus between the sedentary group and the control group identified by the LEfSe analysis were compared in the male and female subjects and the male subjects to explore the impact of gender factor on the gut microbiota. As shown in Figure S4, there were 10 differential genera that were common in the male and female subjects and the male subjects, 4 special genera in the male and female subjects and 4 special genera in the male subjects (Figure S4A). While in ITS sequencing, there were 7 differential genera that were common in the male and female subjects and the male subjects, 5 special genera the male and female subjects and 5 special genera in the male subjects (Figure S4B). The differential bacterial genera between the sedentary group and the control group were similar in the male and female subjects and the male subjects. And most of the differential fungal genera between the control and sedentary group were common in the male and female subjects and the male subjects, suggesting that the impact of gender on the gut microbiota was small in our study.

At the genus level, the bacteria and fungi screened out from the LEfSe analysis were used to distinguish the sedentary and control groups, and the areas under the curve (AUCs) were 0.77 (95% confidence interval (CI): 0.70–0.83), and 0.75 (95% confidence interval (CI): 0.67–0.83), respectively (Figure S4C, D).

### Associations Between the Fecal Microbiota and Clinical Characteristics, and Interactions Between Microbiota

**Fig. 4:**
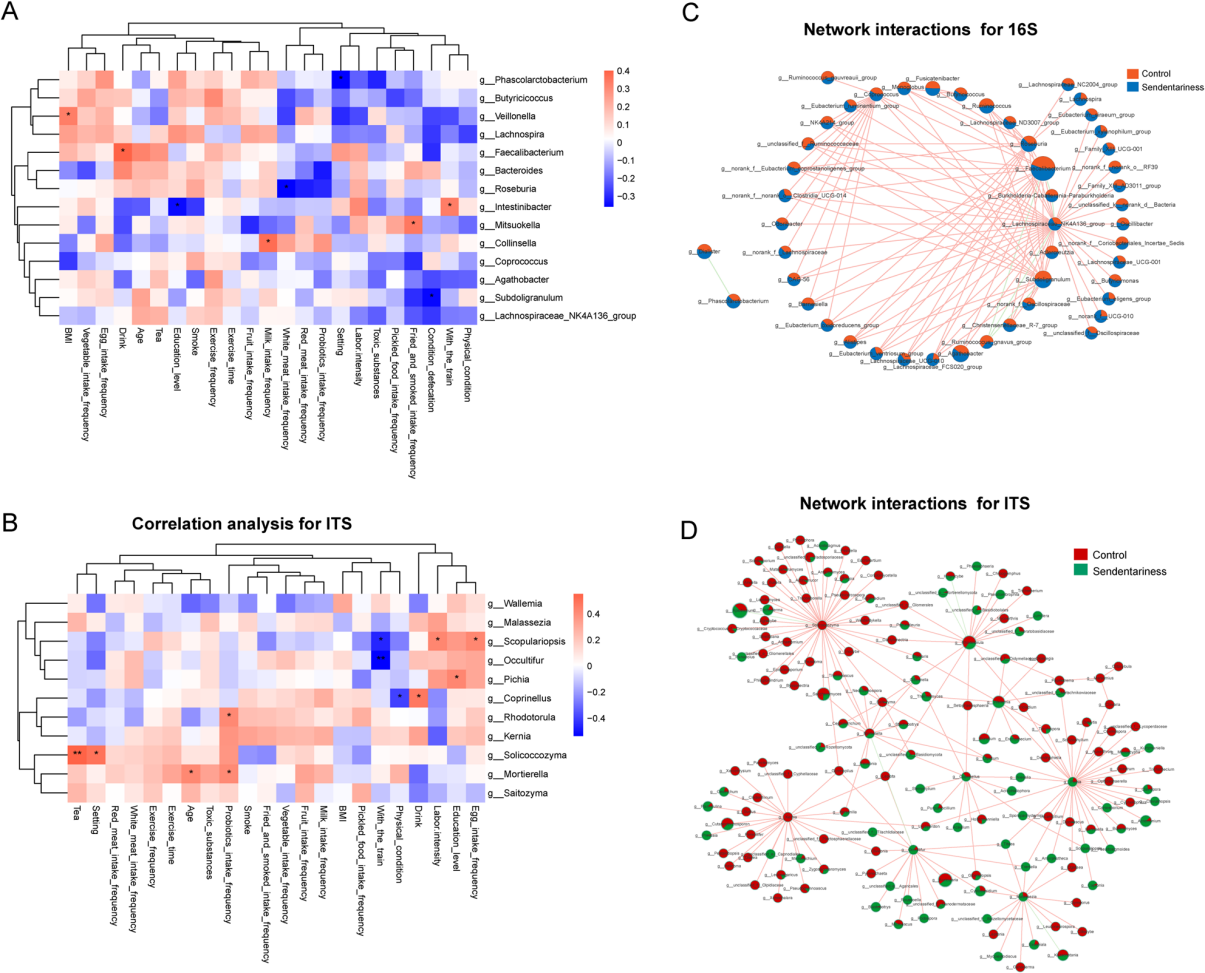
Correlation analysis for the gut microbiota. **a** Correlation analysis between clinical characteristics and bacterial gut microbiota; **b** Correlation analysis between clinical characteristics and fungal gut microbiota; **c** Network interactions between the bacterial gut microbiota; **d** Network interactions between the fungal gut microbiota. Correlation analysis was assessed by Spearman’s. *P < 0.05, **P < 0.01. The orange line means positive regulation, green line means negative regulation. The width of the line represents the correlation coefficient between species. The size of the pie indicates the relative abundance of the genus

We explored the correlation between the fecal microbiota and the clinical characteristics. For the bacterial microbiota, *Veillonella*, *Faecalibacterium*, *Intestinibacter*, *Mitsuokella,* and *Collinsella* were positively correlated with BMI, going out with the train, fried and smoked food intake frequency, and milk intake frequency. *Phascolarctobacterium*, *Roseburia*, *Intestinibacter,* and *Subdoligranulum* were negatively correlated with sitting and the white meat intake frequency (Fig. 4a, b). These data indicate that the gut microbiota was related to many factors, and that a sedentary lifestyle was correlated with *Phascolarctobacterium, Roseburia, Intestinibacter,* and *Subdoligranulum*.

Network interactions between differentially abundant genera from LEfSe analysis and other genera were conducted. Network interactions can vividly show the abundances of genera between the two groups. Through the number of line connections, we identified the genera that interacted the most with other members of the genera. As shown in the Fig. 4c, *Subdoligranulum, Lachnospiraceae_NK4A136_group, Burkholderia-Caballerenia-Paraburkholeria, Faecallbacterium, Roseburi*a, and *Coproooccus* actively interacted with the other genera and were more abundant in the sedentary group. Among these genera, *Faecallbacterium* had the highest abundance, while *Lachnospiraceae_NK4A136_group* was the most active genera *that interacted* with most of the other genera. These two genera may play an important role in the bacterial microbiota. For the fungal microbiota (Fig. 4d), *Pichia, Wallemia, Coprinellus, Occultiful, Malassezia, Rhodotorula, Solicoeeozyma,* and *Kernia* actively interacted with the other genera with many connections, despite their moderate relative abundance. *Solicoeeozyma, Rhodotorula,* and *Kernia* were richer in the control group, while *Pichia, Wallemia, Coprinellus, Occultiful,* and *Malassezia* were richer in the sedentary group, which means that *Solicoeeozyma, Rhodotorula,* and *Kernia* may play an important role in the fungal microbiota of the control group, while *Pichia* and *Malassezia* may play an important role in the fungal microbiota of the sedentary group.

### Function Prediction

**Fig. 5:**
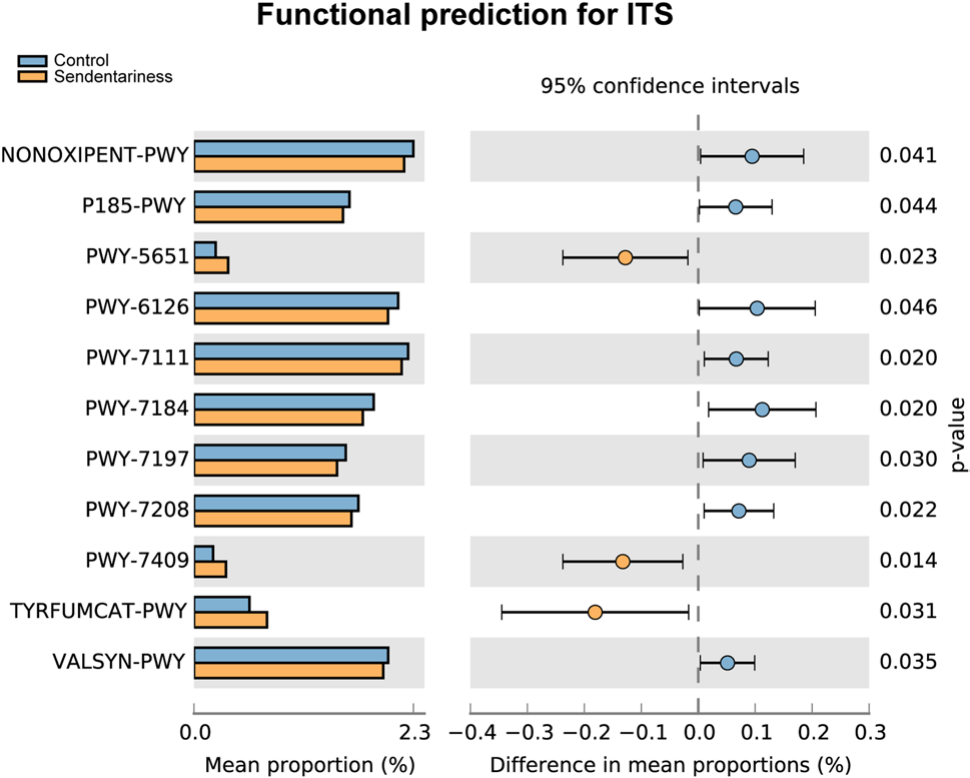
Functional predictions for the gut microbiota. The KEGG pathway with significantly different abundance in the two groups. The left figure shows the abundance ratio of different functional classifications in the two groups. The middle figure shows the difference ratio of functional classification abundance within the 95% confidence interval. The rightmost value is *P* value, and *P* value < 0.05 indicates significant difference. *NONOXIPENT-PWY* pentose phosphate pathway (non-oxidative branch), *P185-PWY* formaldehyde assimilation III (dihydroxyacetone cycle), *PWY-5651* L-tryptophan degradation to 2-amino-3-carboxymuconate semialdehyde, *PWY-6126* superpathway of adenosine nucleotides de novo biosynthesis II, *PWY-7111* pyruvate fermentation to isobutanol (engineered), *PWY-7184* pyrimidine deoxyribonucleotides de novo biosynthesis I, *PWY-7197* pyrimidine deoxyribonucleotide phosphorylation, *PWY-7208* superpathway of pyrimidine nucleobases salvage, *PWY-7409* phospholipid remodeling (phosphatidylethanolamine, yeast), *TYRFUMCAT-PWY* L-tyrosine degradation I, *VALSYN-PWY* L-valine biosynthesis

In the fungal functional prediction, there were significant differences between the two groups (*P* < 0.05) in the functions of the pentose phosphate pathway (non-oxidative branch), formaldehyde assimilation III (dihydroxyacetone cycle), superpathway of adenosine nucleotides de novo biosynthesis II, pyruvate fermentation to isobutanol (engineered), pyrimidine deoxyribonucleotide de novo biosynthesis I, pyrimidine deoxyribonucleotide phosphorylation, superpathway of pyrimidine nucleoside salvage and L-valine biosynthesis, which were all decreased in the sedentary group. The functions of L-tryptophan degradation to 2-amino-3-carboxymuconate semialdehyde, phospholipid remodeling (phosphatidylethanolamine, yeast) and L-tyrosine degradation I were increased in the sedentary group (Fig. 5).

## Discussion

The decrease in the diversity of the gut bacterial microbiota is associated with a variety of acute and chronic diseases [10]. Moreover, studies have suggested that gut fungal microbiota imbalance plays an important role in colorectal, oral, and pancreatic tumors [17], alcoholic fatty liver disease [9], irritable bowel syndrome [18], and inflammatory bowel disease [19]. Many factors affect the diversity, composition, and function of the gut microbiota, as mentioned above. Microbial communities with higher diversity are more stable and more resistant to pathogenic invasions, which is beneficial for host health [2, 20]. Physical exercise improves the prognosis of patients with type 2 diabetes [21], coronary artery disease [22], peripheral artery disease and obesity [23] by increasing the amount of beneficial bacteria and microbial diversity. In contrast, a sedentary lifestyle decreases microbial diversity [12], leading to an increasing incidence of chronic diseases and all-cause mortality [13, 14]. Physical exercise affects the gut microbiota by modulating neuroimmunity and host metabolism. The mechanisms include: altering bile acid profiles, increasing SCFA production [24], increasing immunoglobulin A (IgA) [25], reducing the number of immune cells, reducing intestinal transit time [26], improving intestinal barrier dysfunction [27], and creating an anti-inflammatory intestinal environment to reduce bacterial translocation and modulate microbial composition. Understanding the relationship between the gut bacteria and fungi and a sedentary lifestyle may help to prevent disease and lead to the development of treatment strategies.

Our research showed a lower diversity of the gut bacterial and fungal microbiomes in the sedentary group, which is similar to the results of previous studies [12]. However, the community formation of the gut microbiota between the two groups was similar (Fig. 1c, d), which is reasonable because the subjects were all from the same company and lived in similar environments. The dominant phyla of the sedentary group and the control group were Firmicutes, Actinobacteriota, Proteobacteria, and Bacteroidota, and Firmicutes accounting for 70% (Fig. 2a). Our results are highly consistent with previous studies. Earlier studies [19] revealed that the gut microbiota is mainly composed of Firmicutes, Bacteroidetes, Proteobacteria and Actinobacteria. At the phylum level, the Firmicutes accounts for 65%, and the core composition of the gut microbiota contains approximately 20 genera, including unclassified_Ruminococcaceae, unclassified_Lachnospiraceae, unclassified_Hyphomicrobiaceae, *Roseburia, Faecalibacterium, Blautia, Bacteroides,* and *Bifidobacterium*.

Previous studies have achieved inconsistent results regarding the changes in composition induced by a sedentary lifestyle and exercise because of the difficulties in controling the impact of diet, the environment, and other factors on the gut microbiota. There were significant differences in the relative abundances of some microbiota taxa between the two groups in our study. At the phylum level, the sedentary group had a higher abundance of *Firmicutes* and a comparable abundance of *Bacteroidota,* that is a higher *Firmicutes/Bacteroidota* ratio and a lower *Bacteroidetes/Firmicutes* ratio. This is similar to the report by Denou et al. [28], which demonstrated that high intensity exercise training increases the *Bacteroidetes/Firmicutes* ratio of the mouse distal gut and the fecal microbiota. Our results are partly similar to those of previous studies. Castellos et al. [29], observed that the dominant microbiota of sedentary individuals were from the *Bacteroides* and *Parabacteroides* genera. After a half marathon, the abundance of *Mitsuokella* was increased [30]. Bressa and colleagues found higher abundances of *Barnesiellaceae, Odoribacteraceae, Bifidobacterium, Clostridiales, Turicibacter,* and *Coprococcus* in sedentary women and a lower abundance of *Bacteroidetes* in active women [12]. After exercise intervention, lean individuals have a higher abundance of *Faecalibacterium* and *Lachnospira,* while a higher abundance of *Faecalibacterium* and lower abundance of *Bacteroides* and *Colinsella* were found in obese people [31]. Moreover, the low-fat, high-complex carbohydrate diet increased the *Prevotella* and decreased the *Roseburia* genera*,* while the Mediterranean diet has a converse impact on *Prevotella* and *Roseburia* [32]. Participants in each research come from all over the world and have different dietary structures. Therefore, differences between our results and previous studies may be partly related to the living environment and dietary factors of the subjects.

We also analyzed the composition and differences in the gut fungal microbiota. Consistent with the bacterial microbiota, no significant difference was found in the community formation of the gut fungal microbiota of the two groups (Figure S3 B). Studies [33] have identified gut fungal communities, mainly including *Ascomycota, Basidiomycota, Candida, Zygomycota, Malassezia, Cladosporium,* and *yeast* from the *Dipodascaceae* family. Another study showed that the dominant fungal microbiota of healthy volunteers included *Saccharomyces cerevisiae, Candida albicans, Penicillium,* and *Debaryomyces,* covering 82% of the total numbers [33]. Harry Sokol et al. reported [19] that the most abundant fungal microbiota in healthy subjects and IBD patients were the *Saccharomyces, Debaryomyces, Penicillium, Kluyveromyces,* and *Candida* genera from the Ascomycota and Basidiomycota phyla*.* Therefore, our research results are similar to those of previous studies.

It is difficult to assess the relationship between the fungal community and the host due to the low abundance and large individual differences of the fungal community. Research on the relationship between the gut fungal microbiome and a sedentary lifestyle is still lacking. Our LEfSe analysis of ITSs revealed that the sedentary group had a higher abundance of *Sordariomycetes, Hypocreales, Occultifur_sp, Occultifur, Coprinellus, Scopulariopsis,* and *Malassezia_yamatoensis.* Aleksander Mahnic and colleagues found that more frequent physical activity was related to higher fungal diversity and a lower abundance of *S. cerevisiae* [33]. In our study, the fungal microbiome of the sedentary group was significantly different from that of the control group, and several microbiota constituents were significantly related to activity and sedentary status. A sedentary lifestyle can affect the fungal microbiome despite the small proportion of fungi, and we still need to pay attention to its relationship with diseases and health status.

It is a common phenomenon that information about the composition and diversity is not enough to comprehensively assess the relationship between the microbiota and the host. Research on microbial functions should be considered. Previous studies have mainly focused on the metabolic function of the gut bacterial microbiota induced by exercise [2, 31], and information about the metabolic function of the fungal microbiome is lacking. We found that a sedentary lifestyle decreased the pentose phosphate pathway (non-oxidative branch) and nucleic acid and amino acid biosynthesis and changed the phospholipid metabolism of the gut fungi. There was more L-tryptophan degradation to 2-amino-3-carboxymuconate semialdehyde, more phospholipid remodeling (phosphatidylethanolamine, yeast) and more L-tyrosine degradation I in the fungal microbiome of the sedentary group in our study. It was found that tryptophan was converted to 2-amino-3-carboxymuconate semialdehyde, then degraded to pyruvate and acetate, and finally produced intermediate metabolites such as quinolinate and picolinic acid [34]. Quinolinate (quinolinic acid) may promote the progression of various neurodegenerative disorders [35] due to its neurotoxic effects [36]. Interestingly, picolinic acid can prevent the neurotoxic effects of quinolinic acid [37] by its immunomodulatory properties [38], implying that an imbalance in these metabolites may contribute to disease. In addition, the microbial metabolism of L- tyrosine also can regulate host immunity. The latest research shows that the microbial metabolism of L-tyrosine improves allergic airway inflammation in mice [39]. In other words, the metabolites of the gut fungal microbiota can regulate host immunity.

Phosphatidylethanolamine (PE) is a normal content of the capsules of most of bacteria and archaea, which is synthesized by phosphatidylserine (PS) decarboxylase 1 (Psd1p). PS and PE can modulate the virulence of fungi [40], bacteria, and parasites, as well as autophagy and longevity [41]. Previous studies have shown that fungal PS decarboxylases (PSDs) play a critical role in cell survival, division, and virulence [40]. The phospholipid remodeling (phosphatidylethanolamine, yeast) of the gut fungi in the sedentary group was higher than that in the control group in our study, indicating that a sedentary lifestyle may increase phospholipid remodeling and phosphatidylethanolamine synthesis. It may increase the survival, division, virulence, and longevity of the gut microbiota. However, the interaction between the gut environment and the microbiome is complex and requires further research.

The pentose phosphate pathway (PPP) non-oxidative branch plays an important role in providing precursors for nucleotide and amino acid biosynthesis [42]. The non-oxidative branch metabolizes the intermediates of glycolysis, namely, fructose 6-phosphate, glyceraldehyde 3-phosphate, and sedoheptulose, into 5-phosphate and erythrose 4-phosphate, which are used for the synthesis of nucleic acids and aromatic amino acids. When the requirement for nucleic acids exceeds the requirement for NADPH, the non-oxidative branch of the PPP may promote cell proliferation by providing the nucleic acids [43]. PPP non-oxidative branch-derived nucleotide biosynthesis is critical for synapse formation in neurons [44], which affects the development and metabolism of the brain. Furthermore, the non-oxidative branch of the microbial PPP is related to infection. *Salmonella* contains three transketolases that support the non-oxidative branch of the pentose phosphate pathway [45]. Bacterial sedoheptulose 7-phosphate isomerase converts sedoheptulose 7-phosphate produced by the non-oxidative branching pathway of the PPP to the lipopolysaccharide precursor, glycerol-mannose-heptose 7-phosphate which may promote the bacterial infections. Less pentose phosphate pathway (non-oxidative branch) was found in the sedentary group in our functional prediction analysis of the fungal microbiota, which may have complex and multiple effects on the host.

Our research has several highlights and significance. Previous studies have mainly focused on the gut bacterial microbiota and have paid less attention to the fungal microbiota. We have shown that a sedentary lifestyle changes the compositions and functions of both the gut bacterial and fungal microbiota. In addition, subjects were people with different occupations from the same company, which reduces the impact of environmental and geographical factors on the gut microbiota. Moreover, functional prediction analysis revealed that the potential mechanism of a sedentary lifestyle affects the gut microbiota probably by changing the pentose phosphate pathway (non-oxidative branch) and the nucleic acid, amino acid, and phospholipid metabolism of fungi. However, the research subjects were all employees from the China Railway Corporation, so the population and occupation types should be expanded in the future. Males represented the majority of the subjects in this study, and we need more information about females to compare the effects of a sedentary lifestyle on men’s and women’s gut microbial composition and function. In addition, further studies, such as molecular biology research, are needed to validate our findings regarding the functional prediction of the gut microbiota.

## Conclusion

A sedentary lifestyle reduces the diversity of the gut bacterial microbiome and fungal microbiome, changing the microbial composition. Our results showed that a sedentary lifestyle may affect the predictive functions of the microbiomes by changing the pentose phosphate pathway (non-oxidative branch), nucleic acid and amino acid biosynthesis and phospholipid metabolism of fungi. Further experimental work is required to validate these findings.

### Supplementary Information

Below is the link to the electronic supplementary material.

Supplementary file1 (TIF 18943 kb)
Figure S1. Relative abundance of the gut microbiota. A, Rank-abundance curves for the bacterial gut microbiota on the genus level; B, Rank-abundance curves for the fun gut microbiota on the genus level; C, Number of genera for the bacterial gut microbiota; D, Number of genera for the fungal gut microbiota.

Supplementary file2 (TIF 275 kb)
Figure S2. Venn analysis on ASV level for the gut microbiota. A, Venn analysis on ASV level for the bacterial gut microbiota; B, Venn analysis on ASV level for the fungal gut microbiota. Different groups in the figure are represented by different colors, and the numbers in the figure represent specific or common ASV numbers. The overlapping region represents the number of ASVs common to different groups, while the non-overlapping region represents the number of ASVs unique in each group.

Supplementary file3 (TIF 18842 kb)
Figure S3. The abundance of different species in LEfSe analysis. A, The abundance of the bacterial gut microbiota for two group in genus level; B, The abundance of the fungal gut microbiota for two group in genus level.

Supplementary file4 (TIF 1066 kb)
Figure S4. Comparison of differential bacterial genera identified by the LEfSe analysis between the sedentary group and the control group in male&female subjects and the male subjects. A, Venn analysis on differential bacterial genera for male&female subjects and the male subjects; B, Venn analysis on differential fungal genera for male&female subjects and the male subjects. Different subjects in the figure are represented by different colors, and the numbers in the figure represent specific or common differential genera numbers. The overlapping region represents the number of differential genera that were common in different subjects, while the non-overlapping region represents the number of differential genera that were unique in each subject group. C, Difference in bacterial gut microbiota was used to distinguish the two groups; D, Difference in fungal gut microbiota was used to discriminate the two groups.

## Data Availability

The data are available in National Center for Biotechnology Information (NCBI) under accession PRJNA 761547.
