# Supplementary figures and images for: A Sedentary Lifestyle Changes the Composition and Predicted Functions of the Gut Bacterial and Fungal Microbiota of Subjects from the Same Company
Source: Curr Microbiol. 2023 Oct 13;80(12):368. doi: 10.1007/s00284-023-03480-0 (PMC10575810; doi:10.1007/s00284-023-03480-0)

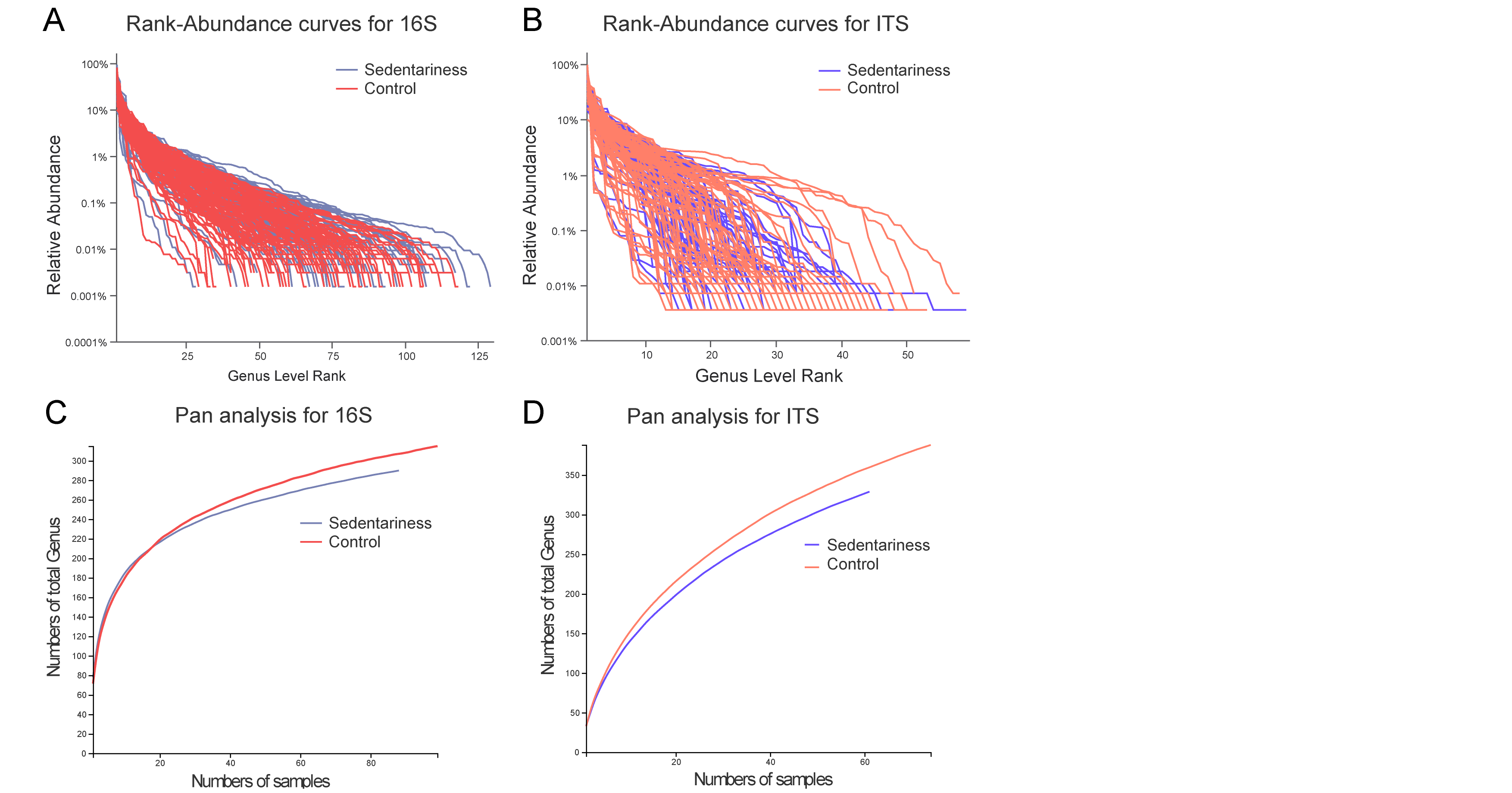

Supplement: Supplementary file 1 — Supplementary file1 (TIF 18943 kb) Figure S1. Relative abundance of the gut microbiota. A, Rank-abundance curves for the bacterial gut microbiota on the genus level; B, Rank-abundance curves for the fun gut microbiota on the genus level; C, Number of genera for the bacterial gut microbiota; D, Number of genera for the fungal gut microbiota. [file 284_2023_3480_MOESM1_ESM.tif]

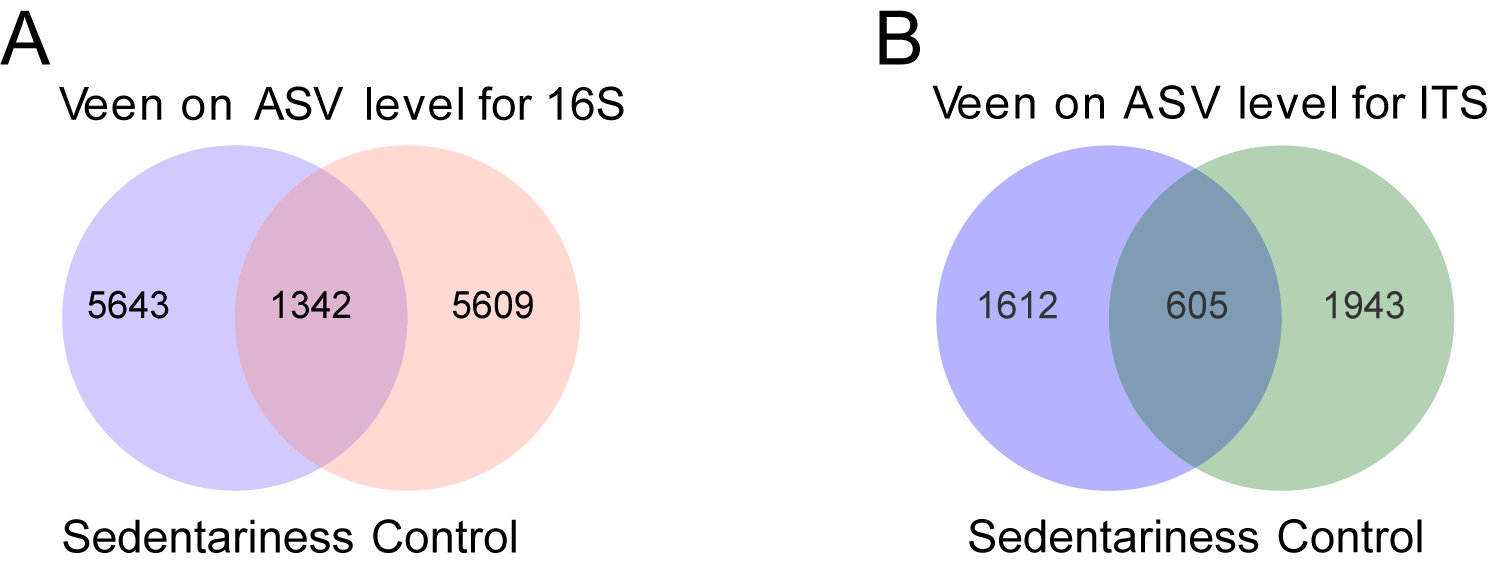

Supplement: Supplementary file 2 — Supplementary file2 (TIF 275 kb) Figure S2. Venn analysis on ASV level for the gut microbiota. A, Venn analysis on ASV level for the bacterial gut microbiota; B, Venn analysis on ASV level for the fungal gut microbiota. Different groups in the figure are represented by different colors, and the numbers in the figure represent specific or common ASV numbers. The overlapping region represents the number of ASVs common to different groups, while the non-overlapping region represents the number of ASVs unique in each group. [file 284_2023_3480_MOESM2_ESM.tif]

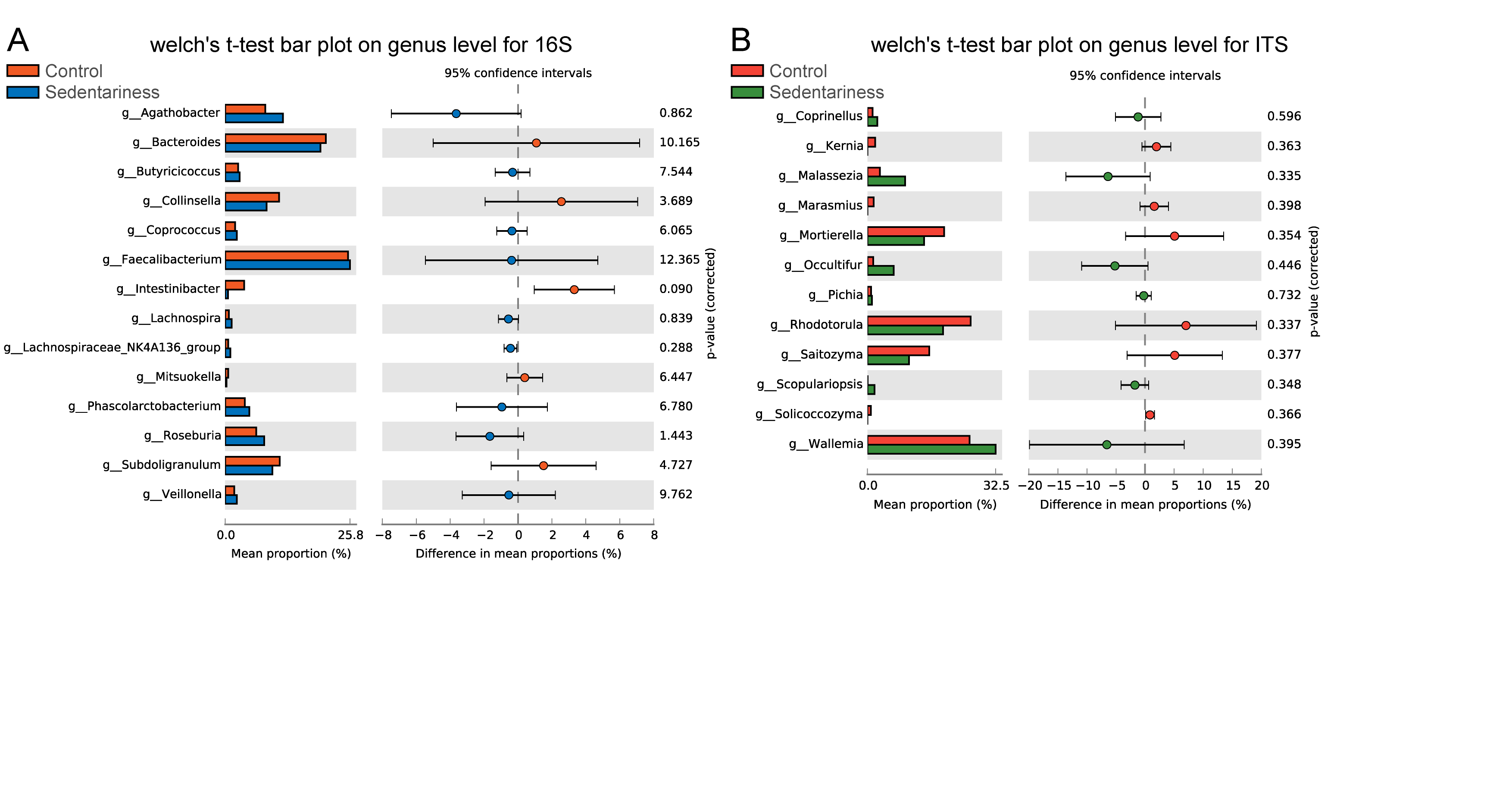

Supplement: Supplementary file 3 — Supplementary file3 (TIF 18842 kb) Figure S3. The abundance of different species in LEfSe analysis. A, The abundance of the bacterial gut microbiota for two group in genus level; B, The abundance of the fungal gut microbiota for two group in genus level. [file 284_2023_3480_MOESM3_ESM.tif]

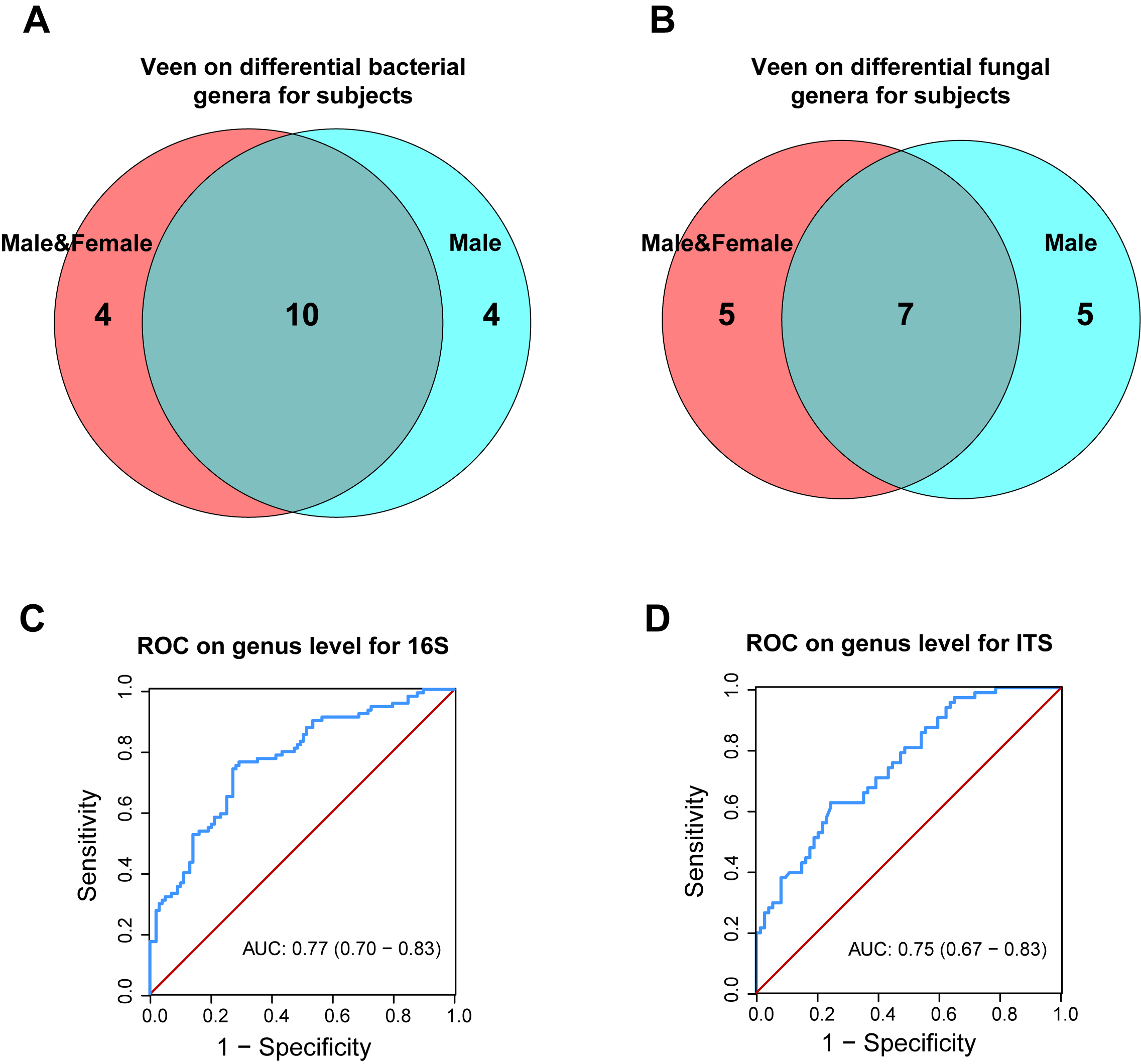

Supplement: Supplementary file 4 — Supplementary file4 (TIF 1066 kb) Figure S4. Comparison of differential bacterial genera identified by the LEfSe analysis between the sedentary group and the control group in male&female subjects and the male subjects. A, Venn analysis on differential bacterial genera for male&female subjects and the male subjects; B, Venn analysis on differential fungal genera for male&female subjects and the male subjects. Different subjects in the figure are represented by different colors, and the numbers in the figure represent specific or common differential genera numbers. The overlapping region represents the number of differential genera that were common in different subjects, while the non-overlapping region represents the number of differential genera that were unique in each subject group. C, Difference in bacterial gut microbiota was used to distinguish the two groups; D, Difference in fungal gut microbiota was used to discriminate the two groups. [file 284_2023_3480_MOESM4_ESM.tif]
